# Setting-Up a Rapid SARS-CoV-2 Genome Assessment by Next-Generation Sequencing in an Academic Hospital Center (LPCE, Louis Pasteur Hospital, Nice, France)

**DOI:** 10.3389/fmed.2021.730577

**Published:** 2022-01-11

**Authors:** Paul Hofman, Olivier Bordone, Emmanuel Chamorey, Jonathan Benzaquen, Renaud Schiappa, Virginie Lespinet-Fabre, Elisabeth Lanteri, Patrick Brest, Baharia Mograbi, Charlotte Maniel, Virginie Tanga, Maryline Allegra, Myriam Salah, Julien Fayada, Jacques Boutros, Sylvie Leroy, Simon Heeke, Véronique Hofman, Charles-Hugo Marquette, Marius Ilié

**Affiliations:** ^1^Laboratory of Clinical and Experimental Pathology, Centre Hospitalier Universitaire de Nice, FHU OncoAge, Université Côte d'Azur, Nice, France; ^2^Hospital-Related Biobank (BB-0033-00025), Centre Hospitalier Universitaire de Nice, FHU OncoAge, Université Côte d'Azur, Nice, France; ^3^Team 4, Institute of Research on Cancer and Aging (IRCAN), CNRS INSERM, Centre Antoine-Lacassagne, Université Côte d'Azur, Nice, France; ^4^Epidemiology and Biostatistics Unit, Centre Antoine-Lacassagne, Université Côte d'Azur, Nice, France; ^5^Department of Pulmonary Medicine and Oncology, Centre Hospitalier Universitaire de Nice, FHU OncoAge, Université Côte d'Azur, Nice, France; ^6^Department of Thoracic H&N Medical Oncology, UT MD Anderson Cancer Center, Houston, TX, United States

**Keywords:** COVID-19, SARS-CoV-2, next-generation sequencing (NGS), variants, Genexus

## Abstract

**Introduction:** Aside from the reverse transcription-PCR tests for the diagnosis of the COVID-19 in routine clinical care and population-scale screening, there is an urgent need to increase the number and the efficiency for full viral genome sequencing to detect the variants of SARS-CoV-2. SARS-CoV-2 variants assessment should be easily, rapidly, and routinely available in any academic hospital.

**Materials and Methods:** SARS-CoV-2 full genome sequencing was performed retrospectively in a single laboratory (LPCE, Louis Pasteur Hospital, Nice, France) in 103 SARS-CoV-2 positive individuals. An automated workflow used the Ion Ampliseq SARS-CoV-2 panel on the Genexus Sequencer. The analyses were made from nasopharyngeal swab (NSP) (*n* = 64) and/or saliva (*n* = 39) samples. All samples were collected in the metropolitan area of the Nice city (France) from September 2020 to March 2021.

**Results:** The mean turnaround time between RNA extraction and result reports was 30 h for each run of 15 samples. A strong correlation was noted for the results obtained between NSP and saliva paired samples, regardless of low viral load and high (>28) Ct values. After repeated sequencing runs, complete failure of obtaining a valid sequencing result was observed in 4% of samples. Besides the European strain (B.1.160), various variants were identified, including one variant of concern (B.1.1.7), and different variants under monitoring.

**Discussion:** Our data highlight the current feasibility of developing the SARS-CoV-2 next-generation sequencing approach in a single hospital center. Moreover, these data showed that using the Ion Ampliseq SARS-CoV-2 Assay, the SARS-CoV-2 genome sequencing is rapid and efficient not only in NSP but also in saliva samples with a low viral load. The advantages and limitations of this setup are discussed.

## Introduction

The COVID-19 pandemic spread across the globe since the beginning of 2020 and still progresses with different waves of infections in all continents (1, 2). Since March 2020, multiple SARS-CoV-2 variants emerged with a large number of mutations, notably in the S protein (3–7). These have been detected in different geographical regions being responsible for the continuous COVID-19 dissemination (8–12). The control of the COVID-19 pandemic requires tremendous efforts to allow widespread screening for SARS-CoV-2 in the general population and to enable worldwide vaccinations. Moreover, these actions must include the possibility to rapidly and massively detect the known viral variants SARS-CoV-2 and quickly define the emergence of novel variants that arise throughout the pandemic (13). Some of these variants are considered by the World Health Organization (WHO) as variants of concern (VOC) because of their potential higher risk to human health (https://www.who.int/csr/don/31-december-2020-sars-cov2-variants/en/, accessed May 6, 2021). Other variants are classified as variants under investigation (since these variants are characterized by some mutations associated with an amino acid replacement which can be responsible for different clusters). Finally, variants under monitoring (VUM) are currently not associated with virological, epidemiological, or clinical data associated with their potential risk to human health (https://www.who.int/csr/don/31-december-2020-sars-cov2-variants/en/, accessed May 6, 2021).

The D614G point-mutation in the Spike protein of SARS-CoV-2 rapidly became the most widespread variant of SARS-CoV-2 at the initial phase of the COVID-19 pandemic (5, 6, 14, 15). A series of other mutations were then identified, allowing the classification of several SARS-CoV-2 lineages and many other mutations will certainly emerge. In this context, the rapid emergence of different variants needs to be assessed in a short time, to evaluate their contagiousness, their association with more severe disease and with possible impact on vaccine efficacy (16–20).

Randomness and natural selection usually determine the fact of newly arising mutations (21). Other potential mechanisms, such as chance events, persistent infection in the immunocompromised host, host shifts, or mutations affecting the proofreading function could also drive viral evolution (22, 23). Indeed, the sudden emergence of new variants which are initially unknown or not detected in certain countries can be associated with a rapid progression of infected clusters of individuals, notably in some populations (at school and universities, hospitals, retirement homes, enterprises, etc.) (24–27). In this context, screening tests using reverse transcription (RT)-PCR may miss these new variants (28) and consequently, the surveillance of the population of the different variants by genomic sequencing is crucial to detect the emergence of new variants and their expansion in a given country or region (29–31). The genomic sequencing should allow not only to do a sustained epidemiological record of the COVID-19 disease in a country but also to start over a short span of time, new health-protection measures according to the detected variant. It is not excluded that the future care of individuals who could be infected by these new variants would be associated with a personalized treatment, and thus that some populations could benefit from vaccine strategies targeting some specific variants (31). In this regard, complete genomic sequencing of the SARS-CoV-2 needs to be performed with flexible and easy-to-use technologies, allowing to get rapid results and therefore accessible to a large number of laboratories. In this regard, complete genome sequencing should be sensitive enough to get robust results even on samples obtained in the community from asymptomatic patients with low viral loads and should not be restricted to hospital samples obtained in patients with high viral loads. Moreover, this sequencing approach should be set up for daily results from several samples analyzed simultaneously.

We report here the introduction of SARS-CoV-2 genomic sequencing in daily practice using the next-generation sequencing (NGS) approach in a single hospital center (LPCE, Louis Pasteur Hospital, Nice, France) with a special emphasis on reducing turn-around-time. We show that this approach allows for each run, a rapid and easy daily analysis of 15 individuals and that this approach can be effective on both nasopharyngeal swabs (NSPs) and saliva samples. The advantages and the limitations of this development are then reported.

## Materials and Methods

### Patients and Samples

**Figure 1:**
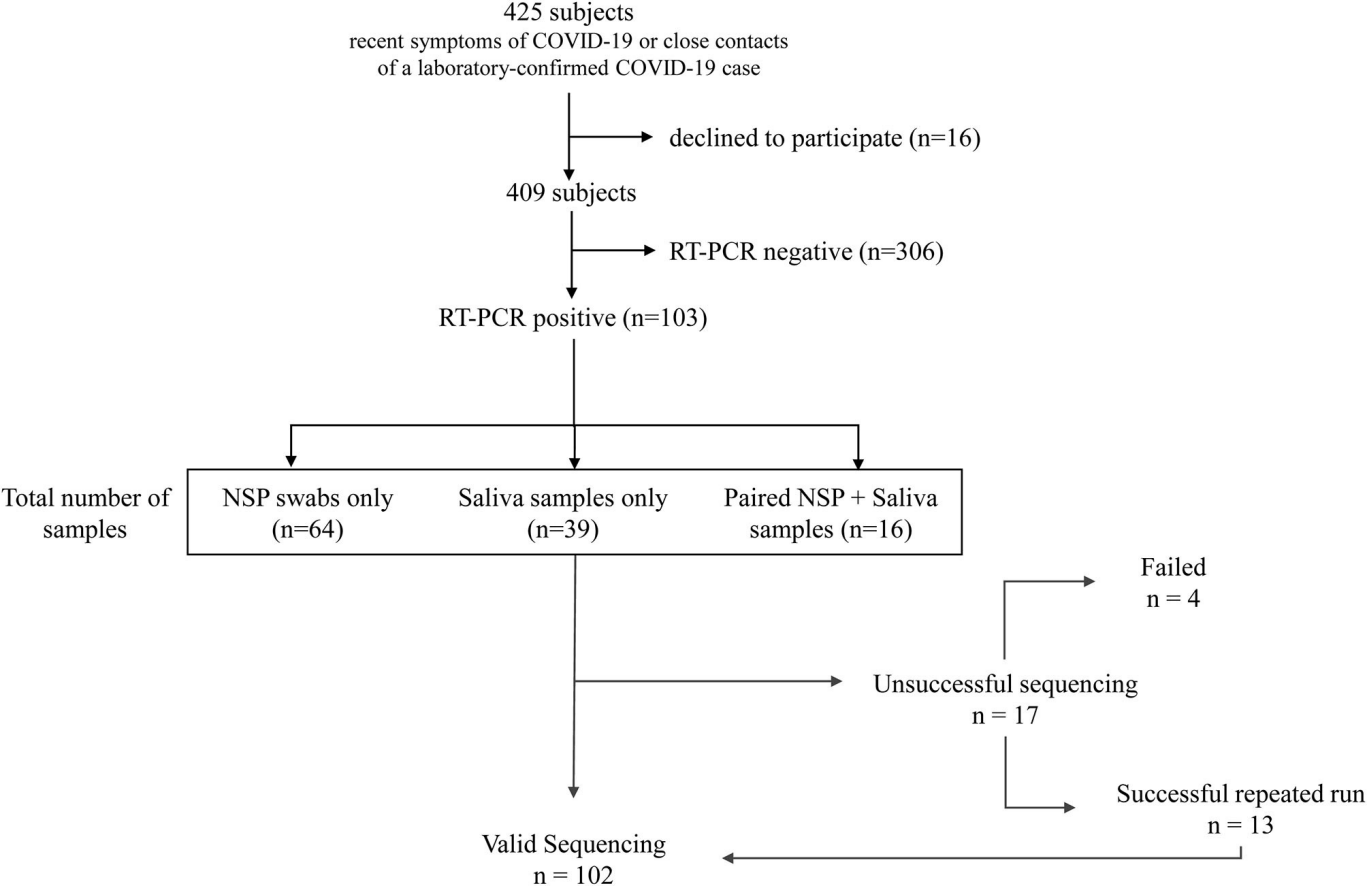
Flowchart of the study.

This study was conducted on a cohort of consecutive volunteers at the Nice-Côte d'Azur Metropolis community-based COVID-19 center (Nice, France), accessible for a free screening to the general population (32). Over 22 weeks (from September 21, 2020 to March 8, 2021), 409 NSP and/or salivary samples were collected in subjects referred: (a) by their attending physician because of recent (≤2 weeks) symptoms of COVID-19 or (b) by the contact tracing staff of the French public health insurance, since they were considered as close contacts of a laboratory-confirmed COVID-19 case. All patients signed informed consent to participate in this study. Only patients with a positive RT-PCR IDYLLA SARS-CoV-2 test were retrospectively included in this study (Figure 1). Specimen included NSP swabs (*n* = 64) and/or saliva (*n* = 39) samples. Moreover, matched NSP swabs and saliva samples were obtained from 16 COVID-19 positive patients, included in the 103 patients cohort, as described previously (33). For initial COVID-19 detection, we used the IDYLLA SARS-CoV-2 kit on the Idylla platform (Biocartis, Mechelen, Belgium) as previously reported (33).

All samples were stored at −80°C at the University Côte d'Azur COVID-19 Biobank (BB-0033-00025, Louis Pasteur Hospital, Nice, France) before their analysis (34). The sponsor of the study was the Center Hospitalier Universitaire de Nice. The agreement for the study of the Institutional review board Sud Méditerranée V was obtained on April 22, 2020 (registration # 20.04014.35208). SHAM liability insurance (No. 159087). The study is registered on ClinicalTrial.gov (NCT04418206).

### SARS-CoV-2 Detection and Genome Sequencing

All samples have been subjected to one freeze-thaw cycle. Viral RNA was extracted with the MagMAX™ Viral/Pathogen Nucleic Acid Isolation Kit (#A42352, Applied Biosystems, Foster City, CA, USA) using the KingFisher™ Duo Prime Purification System (Thermo Fisher Scientific, Waltham, MA, USA).

To measure viral RNA copy number, PCRs were carried with the TaqPath™ 1-Step RT-qPCR Master Mix (#A15299, Thermo Fisher Scientific, Waltham, MA, USA) and TaqMan™ 2019nCoV Assay Kit v1 (#A47532, Thermo Fisher Scientific, Waltham, MA, USA) using the 7500 Fast real-time PCR System (Applied Biosystems). The copy numbers were calculated based on the Ct values, obtained by qPCR for the S, N, and ORF1ab targets, which were translated into the correspondence table in the quick reference “Ion Ampliseq SARS CoV2 research panel” (Thermo Fisher Scientific, Waltham, MA, USA) (13).

Sequencing analyses were performed on the Genexus platform (Thermo Fisher Scientific, Waltham, MA, USA) using the Ion AmpliSeq RNA Custom SARS-CoV-2 kit (Thermo Fisher Scientific, Waltham, MA, USA), which includes two pools of 237 amplicons of 125–275 bp, covering the entire genome of SARS-CoV-2. For each run, 15 samples from the individuals and internal control were used. The fastq files were quality filtered and read mapped with the SARS-CoV-2-Pangolin and “COVID19AnnotateSnpEff” automatic plugins (Thermo Fisher Scientific, Waltham, MA, USA), against the reference genome from Wuhan (GenBank accession number NC_045512.2), to achieve the complete viral genome sequences. The sequencing data were deposited at the European Nucleotide Archive (project number PRJEB47330; https://www.ebi.ac.uk/ena/browser/view/PRJEB47330?show=xrefs).

The criteria to define a valid sequencing or a complete failure were: (i) the number of reads higher than 1 million, (ii) < 1% of unknown nucleotides in the sequence, and (iii) the average depth higher than 1,000. After two re-runs without fulfilling the validity criteria, the result was considered as failed.

### Statistical Analysis

Continuous variables are presented as means (± SD) or median and range; categorical variables as numbers and percentages. Links between variables two by two are evaluated using *t*-test, Wilcoxon test, Spearman correlation, or Fisher's exact test when indicated. Biological variables were tested with and without logarithm transformation. All statistical analyses are done with R.4.0.3 at a significant threshold of 0.05.

## Results

**Figure 2:**
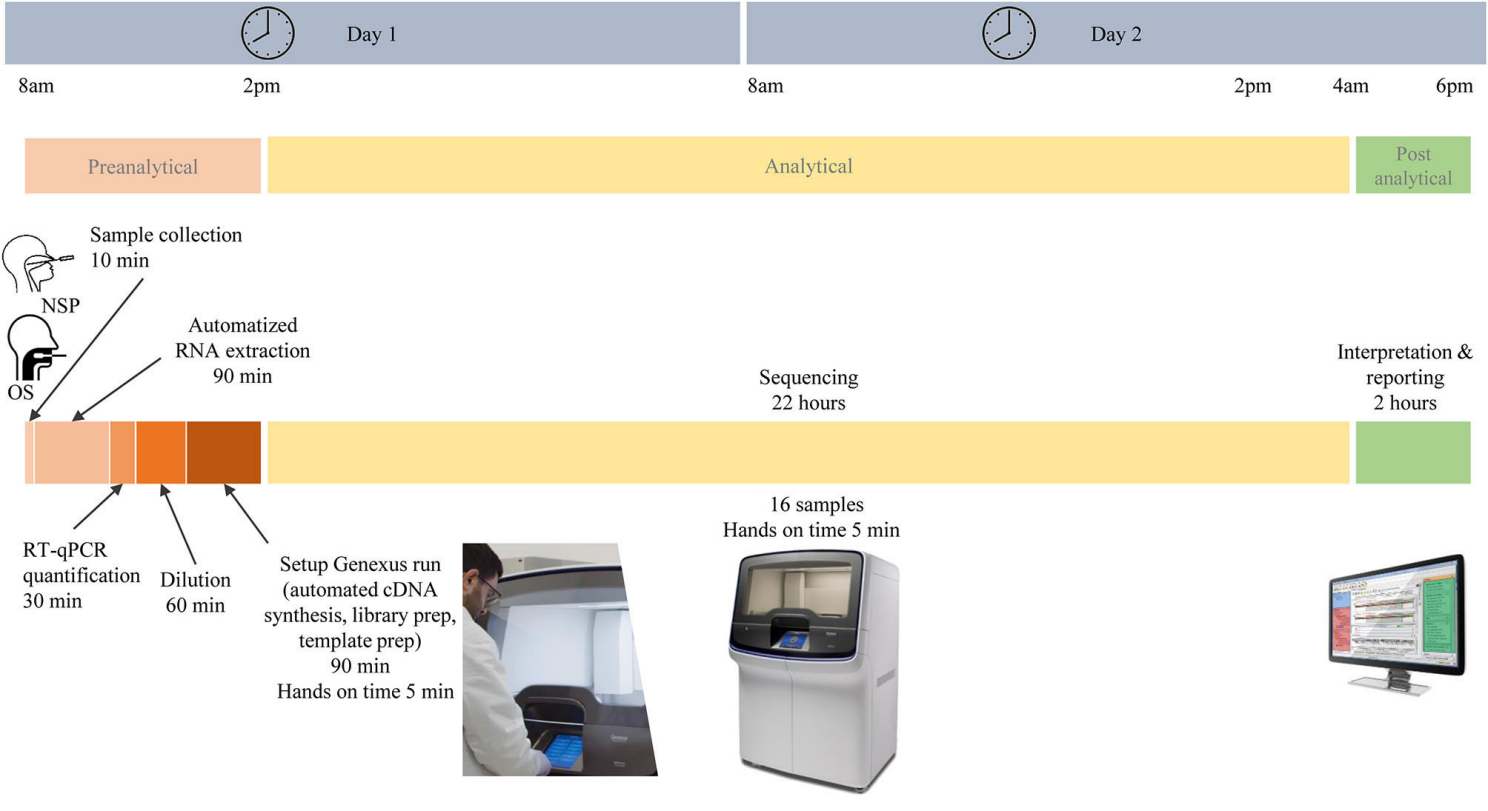
Different pre-analytical, analytical, and post-analytical phases from the selection of samples to the report of SARS-CoV-2 next-generation sequencing.

In total, 103 patients had a positive RT-PCR test. Participants were predominantly women (58/103, 66%), and the mean age (± SD) was 43 ± 15 years. In total, 64 NSP (62%) and 39 saliva samples (38%) were tested on the Genexus platform. In addition, matched NSP swabs and saliva samples were also obtained from 16 SARS-CoV-2 positive individuals. The workflow of samples and the different steps of the preanalytical, analytical, and postanalytical phases are shown in Figure 2. Sequencing results were obtained in ~30 h, including sample collection and RNA extraction until reporting.

**Figure 3:**
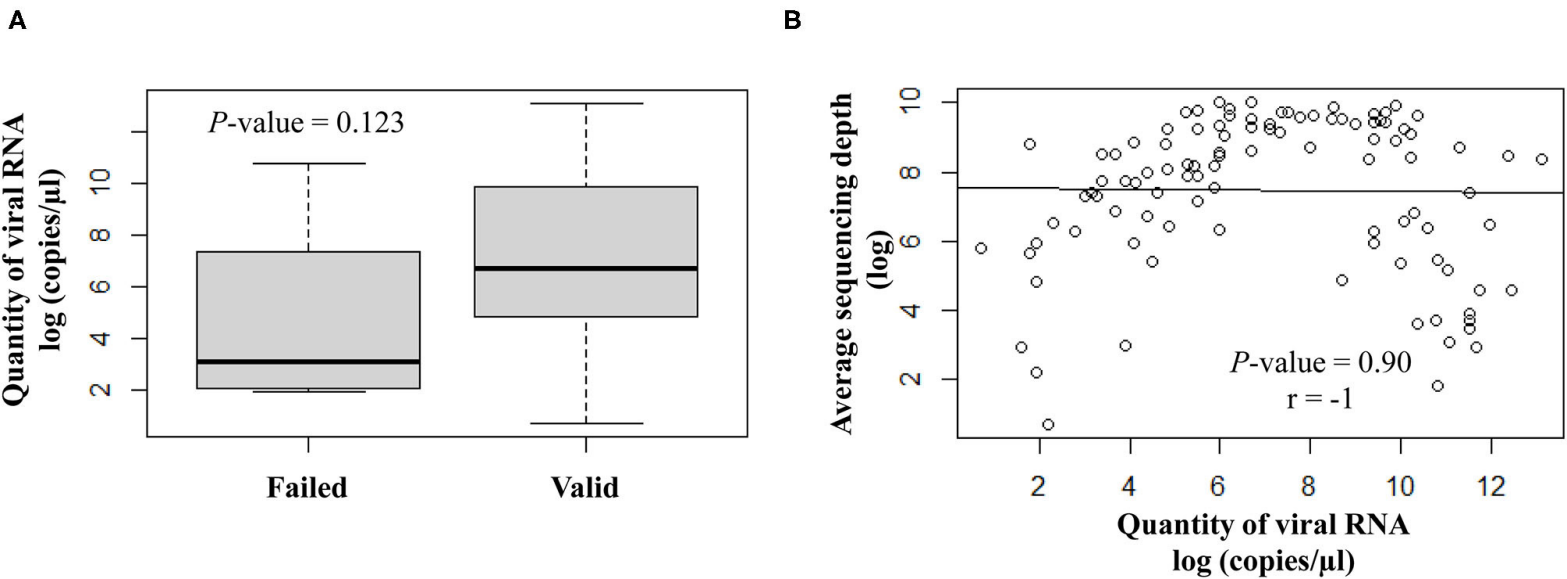
Correlation analysis between the viral RNA load (log copies/μl) and the sequencing results. **(A)** Scatter plot of qualitative variables as per valid or failed sequencing results. **(B)** Quantitative variables are expressed as average sequencing depth.

The quantity of viral RNA varied from 2 to 500,000 copies/μl (mean = 25,593 copies/μl) and from 6 to 16,000 copies/μl (mean = 5,822 copies/μl) for NSP swabs and saliva samples, respectively. The minimum viral RNA quantity to allow sequencing was equal to 2 copies/μl. The quantity of viral RNA did not have a significant impact on the final NGS results (Figure 3).

**Figure 4:**
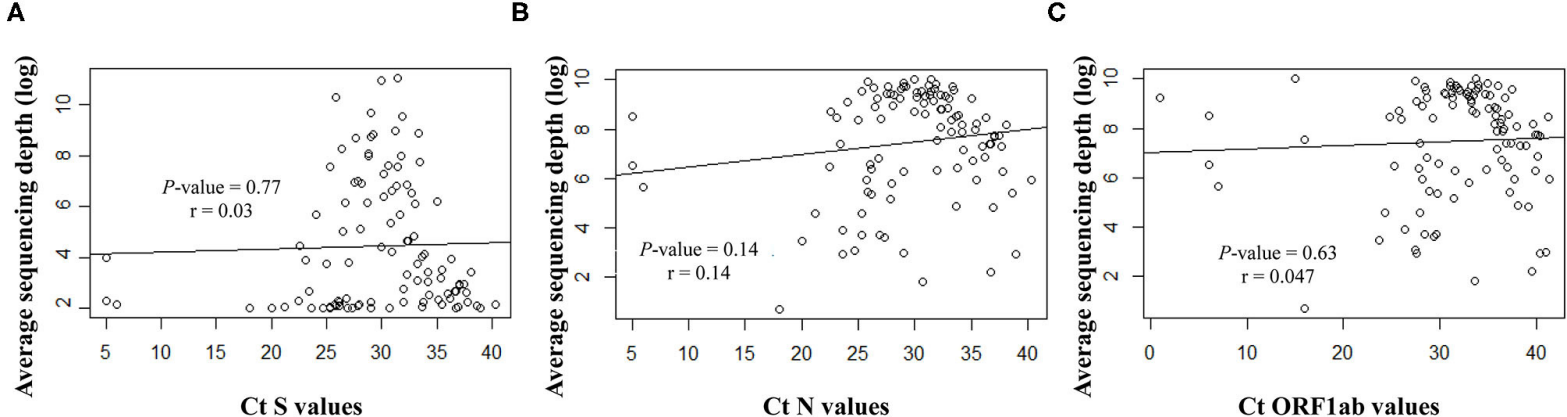
Correlation between the Ct values for the targeted viral gene fragments **(A)** S, **(B)** N and **(C)** ORF1ab, measured with the SARS-CoV-2 Idylla test and the sequencing average depth expressed as logarithmic model.

Overall, the RT-qPCR assay detected SARS-CoV-2 targets with Ct values ranging from 5 to 40.3 (median ± SD, 29.8 ± 6.3). Samples with suitable genome libraries in the NGS protocol included 68/102 (67%) of samples having > 28 Ct values. Concerning the origin of samples, 37/64 (58%) NSP samples had >28 Ct values, whereas 33/39 (85%) saliva samples from asymptomatic subjects had >28 Ct values. No significant difference was observed between the Ct values and the sequencing depth (Figure 4). Samples showed clean mapped reads with an average depth ranging from 1,259 to 22,657 (median, 7,762). Sequencing results and coverage did not appear affected by any difference of the initial Ct values (Figure 4).

After repeated sequencing runs, complete failure of obtaining a valid sequencing result was observed in 4/119 (3%) of samples. Failure was certainly due to degraded RNA combined with a low quantity of viral RNA (e.g., 5, 7, 9, and 50 copies/μl; all were saliva samples from asymptomatic subjects).

**Figure 5:**
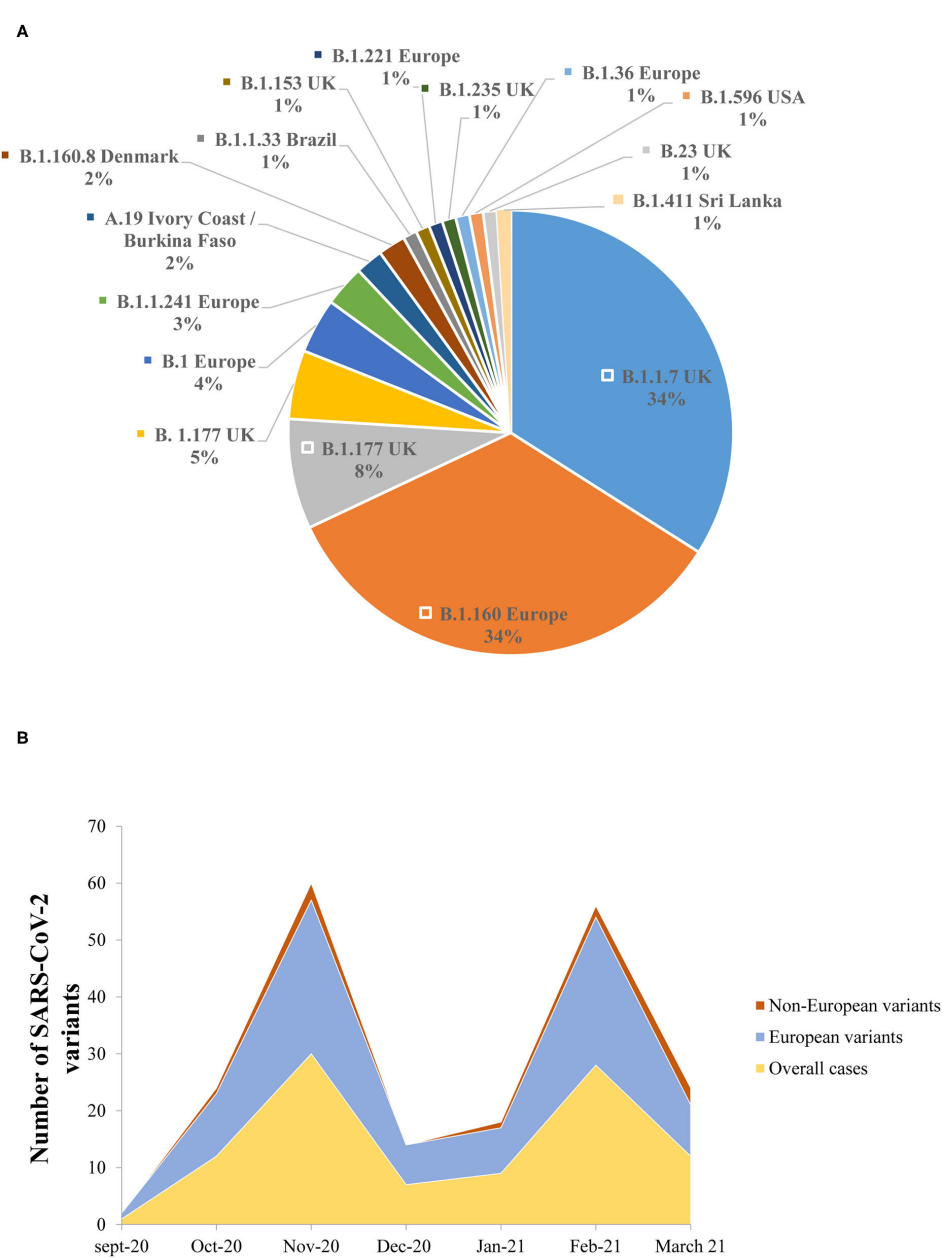
**(A)** The prevalence of the different genomic SARS-CoV-2 variants detected in the study. **(B)** Dynamics of SARS-CoV-2 genomic variants identified in the Nice Metropolitan area (France) from September 21, 2020 to March 18, 2021.

The lineage analysis showed that the majority of the sequences belonged to lineage B, while only 2/102 (2%) sequences belonged to lineage A.19 (Ivory Coast/Burkina Faso lineage) (Figure 5A).

Between September 21, 2020 and October 11, 2020, all the variants isolated were European lineages (Figure 5B). From October 15, 2020 until the end of the study (March 18, 2021), non-European variants were also detected (Figure 5B). It is noteworthy that the B.1.1.7 was first detected on the 8th of February 2021 in the Nice metropolitan area. Other variants detected included, for example, variants from Sri Lanka (B.1.411), Denmark (B.1.160.8), and the USA (B.1.596) (Figure 5B).

Finally, sequencing performed from 16 matched salivary and NSP samples collected at the same time, detected identical variants in 14/16 samples, whereas 2 saliva samples failed to give a valid sequencing run (case #1, viral RNA quantity = 14 copies/μl and average sequencing depth = 13; case #2, viral RNA quantity = 80 copies/μl and average sequencing depth = 1,300).

## Discussion

These preliminary results demonstrated the feasibility of the Ion Ampliseq SARS-CoV-2 Assay to identify known SARS-CoV-2 variants in a daily practice of an academic hospital laboratory. The assay was demonstrated to be easy to use, allowing to get the report of analyses in a reasonable turnaround time in routine patient care (Supplementary Table 1). However, the flow of sequencing is quite limited compared to the flow of screening using the RT-PCR method, since only a maximum of 15 samples (plus a control) can be run each day if a fully automated process is selected (Supplementary Table 1). Therefore, it is helpful to set up an algorithm for rapid integration of SARS-CoV-2 NGS following the screening detection of variants using multiplex RT-PCR. The sensitivity of currently publicly available SARS-CoV-2 real-time RT-PCR panel assays is not perfect and these molecular assays may miss specific variants (28). Thus, a systematic genomic sequencing of NSP and saliva samples positive for RT-PCR SARS-CoV-2 but without complete variants identification using the commercially multiplex test can be realized secondary (8). This two steps analysis may potentially lead to some delay for the final diagnosis, and so be a limitation in the implementation of some health preventive measures to rapidly avoid the dissemination of a newly detected variant with uncertain or well-known infectiousness. Conversely, a front-line SARS-CoV-2 NGS would allow immediate detection of all known variants and to look for new emerging variants at the same time. This latter option can only be devised in a few situations, notably, if the number of samples to be screened is no more than 15 per 24 h. These situations concern the detection of small clusters in determined locations such as the transit zones at airports, some companies, some schools, or retirement houses. Moreover, a couple of patients can show clinical symptoms suggestive of the COVID-19, but the SARS-CoV-2 cannot be detected using RT-PCR in NSP samples. So, SARS-CoV-2 NGS could be done immediately in other biological sources (bronchial aspirates or biopsies, or bronchoalveolar lavages, etc.). Large genome sequencing programs of isolates in the COVID-19 pandemic could provide useful insights into assessing the diagnostic efficacies of new molecular assays (29). Finally, another indication of an immediate genome sequencing may be to look for the SARS-CoV-2 variant in individuals with previous COVID-19 infection and/or having a previous COVID-19 vaccination, but showing new symptoms suggestive of a COVID-19 disease by viral reinfection (22).

Since the beginning of the COVID-19 crisis in China at the end of 2019, numerous SARS-CoV-2 variants emerged in different countries and then disseminated in many regions of the world (1). In this context, an exhaustive surveillance monitoring in France has been set up mainly at the beginning of the three SARS-CoV-2 variants, B.1.17 (VOC 202012/01 or 20B/501Y.V1), B.1.351 (20H/501Y.V2), and P.1 (1.1.281.1). The Indian variant (B.1.617) was detected recently in April 2021 in France in a few individuals too (https://outbreak.info/situation-reports). In fact, the number of different variants has increased during the progression of the pandemic. Variants appeared in one country, then quickly disseminated and spread in different countries and continents. In Europe, notably in France, the first SARS-CoV-2 identified retrospectively in November 2019, was the variant B.1.159 (European variant) (35). Afterward, it was then identified in individuals contaminated during the first wave of the pandemic occurring in France in the first trimester of 2020 (35, 36). This variant was certainly the only one detected in France in individuals until the emergence of the UK variant (B.1.1.7) in England in September 2020 which was rapidly detected in France a few weeks later (12, 24). This latter variant was then rapidly dominant in France early in 2021, and in many countries in Europe and other continents (4, 9, 37). Another variant (B.1.351) was detected in individuals in South Africa in December 2020 (and detected retrospectively in a sample taken on 8 October 2020) (7, 38). A variant from Brazil (B.1.1.28.1) was first detected on the 6th of January 2021 in a traveler returning in Japan coming from Rio de Janeiro (Brazil), found in many individuals in Brazil in the following days, and then rapidly disseminated in many countries (10, 25) (https://virological.org/t/genomic-characterisation-of-an-emergent-sars-cov-2-lineage-in-manaus-preliminary-findings/586; accessed on 3 February 2021). The B.1.351 and B.1.1.281.1 variants were identified in France during the first trimester of 2021. Conversely to the B.1.1.7 variant, these two latter variants are less disseminated in France (around 4% of the different variants) until now (24). One of the variants found in India is the B.1.617 variant, which is characterized by two mutations, E484Q and L452R, known to be associated with increased infectivity and immune escape (39). The WHO reported that the predominant lineage of this variant was first identified in India in December 2020, although an earlier version was spotted in October 2020 (https://www.ecdc.europa.eu/en/publications-data/threat-assessment-emergence-sars-cov-2-b1617-variants). According to GISAID (Global Initiative on Sharing Avian Influenza Data) Database, B.1.617 has been spotted in 18 countries over the world now (https://www.gisaid.org/hcov19-variants/). By the 29 April of 2021, France detected three cases of the B.1.617 variant of the Covid-19 currently sweeping India (11, 27, 40, 41). Finally, many other variants of interest were detected in many countries, such as in the USA, France, and Uganda (11, 27, 40, 41). Taken together, all these variants detection highlight the urgent need to develop large genomic sequencing programs in all countries to be able to immediately identify the onset of a potential new variant having high infectiousness and possible resistance to the different types of immune response developed by current vaccines too (1).

The NGS analysis performed from the samples included in this retrospective series and derived from individuals collected in Nice (France) from September 2020 to March 2021, did not detect South African and Indian variants. The only VOC isolated in our cohort was the B.1.1.7. A couple of other variants (or variants under monitoring) were detected among this population, such as the B.1.596 (USA), the B.1.411 (Sri Lanka), and the A.19 (Ivory Cost/Burkina Faso) variants.

It is noteworthy that results obtained from matched NSP and saliva samples showed excellent concordance. These results open the door for making NGS from saliva samples for SARS-CoV-2 identification. So, the SARS-CoV-2 screening is currently more and more developed using saliva samples instead of NSP swabs, since taking saliva is non-invasive in comparison to NSP swabs and can be the only possibility to get a screening test in certain populations. Our results showed that NGS can be now adapted from saliva for SARS-CoV-2 variant identification.

In our study, variant identification was efficient even for low viral loads (Ct > 28) detected in samples of different origins, including saliva (32, 33). This highlights that using the Ion Ampliseq SARS-CoV-2 Assay run on the Genexus could allow genomic sequencing from samples with a low viral load, notably derived from COVID-19 asymptomatic individuals. The failure rate was low (4%) and was most probably due to degraded RNA (e.g., all were saliva samples from asymptomatic subjects) combined with very low viral loads.

However, our study shows a couple of limitations. First, it is a retrospective study made from a quite low number of individuals. So, this approach needs to be validated in daily practice with the usual workflow of samples routinely registered in a laboratory for SARS-CoV-2 detection. Secondly, the period of inclusion was limited to a 6 months' time duration and did not allow us to isolate some new emerging variants, notably the South African, Brazilian, and Indian variants. So, the Ion Ampliseq SARS-CoV-2 Assay was tested on a limited number of samples from the Nice population almost all affiliated to the Pangolin classification (42). Amplicon-based target enrichment can be impacted by SNP or indels located with primer-annealing regions even if their tiling amplicon designs aim to reduce the impact of such modifications. Moreover, as SARS-CoV-2 may potentially evolve, primers for amplicon-based target enrichment need to be constantly updated.

In conclusion, this work showed the feasibility of rapidly setting up an NGS approach using the Ion Ampliseq SARS-CoV-2 Assay, to routinely identify known SARS-CoV-2 variants in a daily practice of a hospital laboratory. This study demonstrated that only one VOC (B1.1.7) was identified in some individuals included in this period, since all other variants detected were from the VUM category. Moreover, further studies could be developed to demonstrate that the current approach may potentially be of interest in the near future for the identification of some unknown SARS-CoV-2 variants. Therefore, this approach could improve the NGS surveillance to cover emerging SARS-CoV-2 variants, but certainly through panel design improvements. Moreover, it should increase the sensitivity of the SARS-CoV-2 panel to enable an improved limit of detection from various sample types. This could enable more investigators to access NGS to rapidly obtain epidemiological insights with rapid turn-around time, workflow automation, and seamless informatics and data uploading to public SARS-CoV-2 data repositories too.

Importantly, we demonstrated that SARS-CoV-2 genome assessment can be made in different samples, including saliva with low viral load obtained from asymptomatic individuals. Increased genetic sequencing and PCR-based detection of the B.1.1.7 variant, and other variants of SARS-CoV-2 is an action plan not only in Europe but in all continents for setting up a defense against new SARS-CoV-2 variants (43). In this regard, SARS-CoV-2 NGS could be used as a screening method, but according to the clinical presentation, to the urgent need and also to the number of samples registered in daily practice for the RT-PCR screening test.

## Data Availability Statement

The original contributions presented in the study are publicly available. This data can be found at: https://www.ebi.ac.uk/ena/browser/view/PRJEB47330.

## Ethics Statement

The studies involving human participants were reviewed and approved by Sud Méditerranée V. The patients/participants provided their written informed consent to participate in this study.

## Author Contributions

PH, VH, C-HM, and MI contributed to the study design. PH, JBe, OB, VL-F, EL, PB, BM, CM, VT, MA, MS, JF, JBo, SL, SH, VH, C-HM, and MI contributed to data collection and collection of clinical specimens. OB, VL-F, EL, CM, VT, MA, MS, and JF contributed to experiments. EC contributed to the data analysis. PH, EC, C-HM, and MI contributed to the manuscript preparation. All authors contributed to the article and approved the submitted version.

## Funding

This work was supported by Agence Régionale de Santé Provence-Alpes-Côte d'Azur, Conseil Départemental 06 des Alpes Maritimes, Ville de Nice, Métropole Nice Côte d'Azur, Fonds de Dotation AVENI, and from private donators.

## Conflict of Interest

PH received honoraria from Thermo Fisher Scientific for participating in scientific meetings, outside this present work. The remaining authors declare that the research was conducted in the absence of any commercial or financial relationships that could be construed as a potential conflict of interest.

## Publisher's Note

All claims expressed in this article are solely those of the authors and do not necessarily represent those of their affiliated organizations, or those of the publisher, the editors and the reviewers. Any product that may be evaluated in this article, or claim that may be made by its manufacturer, is not guaranteed or endorsed by the publisher.
