# Supplementary material for: Setting-Up a Rapid SARS-CoV-2 Genome Assessment by Next-Generation Sequencing in an Academic Hospital Center (LPCE, Louis Pasteur Hospital, Nice, France)
Source: Front Med (Lausanne). 2022 Jan 11;8:730577. doi: 10.3389/fmed.2021.730577 (PMC8787061; doi:10.3389/fmed.2021.730577)
Supplement: Supplementary file 1 [file Table_1.docx]

**Supplementary Table 1.** Technical and logistic comparison between the main SARS-CoV-2 genome sequencing platforms.

| **Company** | **Thermo Fisher Scientific**  Waltham, MA, USA | **Illumina**  San Diego, CA, USA | **Oxford Nanopore**  Oxford, UK | **MGI**  Shenzhen, CHINA |
| --- | --- | --- | --- | --- |
| Sequencing Platform | Genexus Integrated Sequencer, IonChef System / IonGeneStudio S5 System | NovaSeq, NextSeq, MiSeq | GridIon, Minion, Minion Mk1C | DNBSeq Systems |
| Protocol | Ion AmpliSeq SARS-CoV-2 Research Panel | AmpliSeq SARS-CoV-2 panel | AmpliSeq SARS-CoV-2 panel | Paragon Genomics Amplicon Kit,  Twist Hybrid Capture Kit |
| TAT (Library Prep to Sequencing) (hours) | 24-30 | 42-69 | 7 | 8 hours for sequencing only |
| Automation (Library to Analysis) | Yes | No | No | No |
| Minimum Input requirement (viral copies) | 150 | 1000 | 1000 | 1* |
| Sample Flexibility and Scalability | Yes | No | Yes | No |
| Accessibility | ++++ | ++ | ++++ | + |
| Sample Cost | $-$$$ | $-$$$ | $ | $ |
| SNP Error rate | 0.036% | 0.26-0.51% | 5% | 0.26-0.51% |
